# Clinicopathological characteristics of early gastric cancer with different level of undifferentiated component and nomogram to predict lymph node metastasis

**DOI:** 10.3389/fsurg.2023.1097927

**Published:** 2023-02-13

**Authors:** Chenyu Li, Suling Xie, Dan Chen, Jingwen Zhang, Ning Zhang, Jinchao Mu, Aixia Gong

**Affiliations:** ^1^ Department of Gastroenterology, First Affiliated Hospital, Dalian Medical University, Dalian, China; ^2^ Department of Pathology, First Affiliated Hospital, Dalian Medical University, Dalian, China; ^3^ Department of Geriatric Medicine, First Affiliated Hospital, Dalian Medical University, Dalian, China

**Keywords:** early gastric cancer, lymph node metastasis, mixed type, predicting, nomogram

## Abstract

**Background:**

Few studies showed that mixed type early gastric cancer (EGC) relates to higher risk of lymph node metastasis. We aimed to explore the clinicopathological feature of GC according to different proportions of undifferentiated components (PUC) and develop a nomogram to predict status of lymph node metastasis (LNM) in EGC lesions.

**Methods:**

Clinicopathological data of the 4,375 patients who underwent surgically resection for gastric cancer in our center were retrospectively evaluated and finally 626 cases were included. We classified mixed type lesions into five groups (M1:0% < PUC ≤ 20%, M2:20%<PUC ≤ 40%, M3:40%<PUC ≤ 60%, M4:60%<PUC ≤ 80%, M5:80%<PUC < 100%). Lesions with 0% PUC were classified as pure differentiated group (PD) and lesions with 100% PUC were classified as pure undifferentiated group (PUD).

**Results:**

Compared with PD, LNM rate was higher in group M4 and group M5 (*p* < 0.05 after Bonferroni correction). Differences of tumor size, presence of lymphovascular invasion (LVI), perineural invasion and invasion depth also exist between groups. No statistical difference of LNM rate was found in cases who met the absolute endoscopic submucosal dissection (ESD) indications for EGC patients. Multivariate analysis revealed that tumor size over 2 cm, submucosa invasion to SM2, presence of LVI and PUC level M4 significantly predicted LNM in EGC. With the AUC of 0.899(*P* < 0.05), the nomogram exhibited a good discrimination. Internal validation by Hosmer–Lemeshow test showed a good fitting effect in model (*P* > 0.05).

**Conclusion:**

PUC level should be considered as one of the predicting risk factors of LNM in EGC. A nomogram that predicts the risk of LNM in EGC was developed.

## Introduction

Worldwide, gastric cancer is the fourth leading death caused by cancer (1). Regardless of LNM, tumors confined to the mucosa or submucosa layer are defined as EGC (2). Prevalence of routine endoscopic screening programs under white light imaging together with advances such as magnifying narrow band imaging or magnifying blue laser imaging increased EGC detection rate and accuracy (3, 4). As a consequence, more patients with gastric cancer had their lesions resected at early stage under endoscopy or laparoscopy (5, 6). Endoscopic submucosal dissection (ESD), since its safety and high efficacy, had been accepted as a therapy for resection of EGC with a limited size and very low lymph node metastasis (LNM) risk (2, 7). Since the integrity of stomach is conserved, there is less postoperative syndrome after ESD and postoperative life quality could be improved when compared to surgical resection (8–10). But lymph node dissection and examination couldn`t be performed during ESD procedure, which is one of the limitations. However, when deciding whether to choose ESD for treatment of EGC, incidence of LNM is an important factor considered (11). Histological differentiation, as a crucial factor which relates to the risk of LNM, is considered in indications for ESD (12, 13). Gastric cancer often presents with a mixed histology type even in early stage (14). According to guidelines of Japanese Gastric Cancer Association (JGCA), histological type of cancer tissues could be classified as differentiated type and undifferentiated type (2). Mixed type gastric cancer in our study refers to tumor with a mixed differentiation. Studies around LNM risk in mixed type EGCs are controversial, and there is still no recommended ESD indication for mixed type lesions. Several studies showed that mixed histological type was not associated with a higher LNM risk for EGC patients and ESD indications could be applicable to mixed type EGC (15–18). On the other hand, there were also reports demonstrated that mixed histologic type was more aggressive since it is associated with a higher LNM rate when compared with pure differentiated or pure undifferentiated tumors (19–22). Indications of ESD for EGC had recently been updated (23). According to the latest edition, lesions which met with expanded indications for ESD in the first edition had been integrated into absolute indications. For ratio of differentiated components or undifferentiated components varies in different lesions, we hypothesis that differences of LNM rate together with other clinicopathological features might exist in lesions according to PUC level. To the best of our knowledge, no study had yet focused on this point. In our study, clinicopathological features including LNM status of EGC lesions according to different PUC level was explored, applicability of ESD indications for mixed type EGC was investigated. Afterwards, a nomogram was also developed to predict status of LNM in EGC lesions, which might assist clinicians in choosing more suitable treatment strategy for EGC patients.

## Method

### Patients and variables

We consecutively reviewed clinicopathological data of the 4,375 patients who underwent surgically resection with lymph node dissection for gastric cancer in our hospital between January 2014 to January 2022 and 655 patients diagnosed EGC by postoperative pathology were selected. Exclusion criteria were as follows: (I) Pathological types (neuroendocrine tumor, carcinoma with lymphoid stroma and hepatoid carcinoma) outside the scope of this study (6 cases were excluded). (II) Cases with multiple synchronous cancers (12 cases were excluded). (III) Preoperative endoscopic treatment (2 cases were excluded). (IV) Local recurrence (2 cases were excluded). (V) Patients who had undergone chemotherapy and/or radiation (0 cases were excluded). (VI) Cases with no preoperative endoscopic images or reports (7 cases were excluded). A total of 626 patients with 626 lesions were finally enrolled. Clinical and pathological data including sex, age, tumor location, tumor size, macroscopic type, ulceration, invasion depth, LVI, perineural invasion, histological type, scope of LND (lymph node dissection), number of LND LNM status were collected. Cut value of age was set to 60 (years) as mean age of the cohort was 63.6 (±8.8) years. For tumor size, according to the calculated mean value (2.4 ± 1.3 cm), we graded lesions into small group(≤2 cm) and large group (>2 cm). For tumor location, we classified the lesions into upper, middle or lower 1/3 of stomach (2). Classification of tumor gross morphology were based on Paris endoscopic classification (9). Invasion depth included three following grades: mucosal(M), SM1 (depth of invasion <500 *μ*m) and SM2 (depth of invasion ≥500 μm). Judgement of LND scope involved in this study (D1, D1+, D2) was in accordance with Japanese gastric cancer treatment guidelines (2). Cut value of node dissection number was set to 22 as mean number of the cohort was 22.3 (±9.6).

### Pathological evaluation

All lesions were sliced at intervals of 3 to 5 mm. Lymph nodes (at least 15 nodes were harvested per case) were sliced and stained with hematoxylin and eosin to assessment the presence of LNM. Pathological parameters were evaluated according to guidelines of Japanese Gastric Cancer Association (JGCA) for EGC (2, 23). To assess proportion of undifferentiated components, in this study, the panel of three pathologists examined all slides of all the specimens. Different histological cancer areas of all slides were evaluated under microscope and finally summarized to determine the PUC level.

### Grouping methods

To further investigate differences of clinicopathological characteristics between different PUC levels, we grouped mixed histologic type lesions into five groups (M1:0% < PUC ≤ 20%, M2:20% < PUC ≤ 40%, M3:40% < PUC ≤ 60%, M4:60%<PUC ≤ 80%, M5:80% < PUC < 100%). Together with PD (PUC = 0%) and PU lesions (PUC = 100%), clinicopathological features according to PUC level were explored. According to guidelines of ESD for EGC, tumor differentiation is determined according to its quantitative dominant component (23). Thus in our study, mixed histological type lesions were regrouped into four groups: G1(0% < PUC ≤ 25%), G2 (25% < PUC < 50%), G3 (50% ≤ PUC ≤ 75%) and G4 (75% < PUC < 100%). Compared with PD, ESD indications for differentiated EGC were verified in G1 and G2. Compared with PU, ESD indications for undifferentiated EGC were verified in G3 and G4.

### Statistical analysis

Statistical analyses were conducted by SPSS 26.0 software and R software version 4.2.0. Continuous variables (age and tumor size) were translated into categorical variables. Differences of clinical and pathological features between groups were analyzed by Pearson chi-square or Fisher`s exact test. Variables that were statistically associated with LNM in univariate analysis were then entered into a logistic regression model to investigate independent risk factors. Accuracy of the nomogram was validated by the area under the curve (AUC), concordance-index(C-index) was also applied to estimate nomogram performance. Under Bootstrap method, repetitive sample of the same size were constructed. The sample was used as the training set, and the corresponding unsampled queue was used as the verification set. This performance evaluation was repeated 1,000 times to obtain the calibration curve. Hosmer-lemeshow test was used to calculate the goodness of fit of the model. *P* < 0.05 means the difference is regarded as statistically significant.

## Results

### General clinicopathological characteristics

A total of 626 patients with a total of 626 lesions were finally included in this study. Of the patients, 429(68.5%) were male and 197(31.5%) were female. Incidence of LNM in this study was 11.3% (71 of 626). Forty-eight (7.7%) lesions were in the upper third of the stomach, 156(24.9%) in the middle third, and 422(67.4%) in the lower third. Proportion of PD, mixed and PU histology type in this study was 49.8% (*n* = 312), 30.7% (*n* = 192) and 19.5% (*n* = 122), respectively. Together with the parameters mentioned above, clinicopathological characteristics of the whole cohort were shown in Table 1.

**Table 1:** General data of clinicopathological characteristics.

Variable	626 cases with 626 lesions
Age (years)	
≤60	202 (32.3%)
>60	424 (67.7%)
Sex	
Male	429 (68.5%)
Female	197 (31.5%)
Tumor location	
Upper third	48 (7.7%)
Middle third	156 (24.9%)
Lower third	422 (67.4%)
Tumor size	
≤2 cm	315 (50.3%)
>2 cm	311 (49.7%)
Macroscopic type	
0-I (Protruded)	47(7.5%)
0-IIa (Elevated)	57 (9.1%)
0-IIb (Flat)	67(10.7%)
0-IIc (depressed)	308 (49.2%)
0-III (excavated)	147(23.5%)
Ulcer	
Absence	307 (49.0%)
Presence	319 (51.0%)
Invasion depth	
M	302 (48.2%)
SM1	109 (17.4%)
SM2	215 (34.3%)
LVI	
Absence	546 (87.2%)
Presence	80 (12.8%)
Perineural invasion	
Absence	595 (95.0%)
Presence	31 (5.0%)
Histological type	
PD	312 (49.8%)
Mixed type	192 (30.7%)
PU	122 (19.5%)
LNM	
Negative	555 (88.7%)
Positive	71 (11.3%)
scope of LND	
D1	57 (9.1%)
D1+	131 (20.9%)
D2	438 (70.0%)
Number of LND	
≤22	399 (63.7%)
>22	227 (36.3%)

LND, lymph node dissection.

### Clinicopathological characteristics according to different PUC levels

To further investigate differences of clinicopathological characteristics between different level of PUC, we grouped mixed histologic type lesions in to five groups: M1 (*n* = 26, 4.2%), M2 (*n* = 34, 5.4%), M3 (*n* = 30, 4.8%), M4 (*n* = 41, 6.5%), M5 (*n* = 61,9.7%). Clinicopathological features of the patients based on different level of PUC were shown in Table 2. Distribution of age, sex, tumor location, macroscopic type, presence of ulcer, scope of LND and number of LND in lesions with different PUC levels showed no difference (*p* > 0.05 after Bonferroni correction). Compared with pure undifferentiated lesions, pure differentiated lesions tend to have less presence of perineural invasion (*p* = 0.025 after Bonferroni correction). When compared with PD group, mixed type tumors in M4 were larger (*p* = 0.025), tumors in M5 were more frequently with submucosal invasion to SM2 (*p* < 0.05), and lesions in M4 (*p* < 0.001) or M5 (*p* = 0.035) were prone to LVI (All *p* value were corrected under Bonferroni method).

**Table 2:** Clinicopathological characteristics of early gastric cancer with PUC level.

Variable	PD *n* (%)	M1 *n* (%)	M2 *n* (%)	M3 *n* (%)	M4 *n* (%)	M5 *n* (%)	PU *n* (%)	*p*
Age (years)								0.099
≤60	85 (27.2%)	8 (30.8%)	9 (26.5%)	11 (36.7%)	15 (36.6%)	23 (37.7%)	51 (41.8%)	
>60	227 (72.8%)	18 (69.2%)	25 (73.5%)	19 (63.3%)	26 (63.4%)	38 (62.3%)	71 (58.2%)	
Sex								0.151
Male	229 (73.4%)	17 (65.4%)	22 (64.7%)	21 (70.0%)	26 (63.4%)	42 (68.9%)	72 (59.0%)	
Female	83 (26.6%)	9 (34.6%)	12 (35.3%)	9 (30.0%)	15 (36.6%)	19 (31.1%)	50 (41.0%)	
Tumor location								0.201
Upper third	26 (8.3%)	5 (19.2%)	2 (5.9%)	3 (10.0%)	3 (7.3%)	0 (0.0%)	9 (7.4%)	
Middle third	74 (23.7%)	5 (19.2%)	8 (23.5%)	5 (16.7%)	13 (31.7%)	15 (24.6%)	36 (29.5%)	
Lower third	212 (67.9%)	16 (61.5%)	24 (70.6%)	22 (73.3%)	25 (61.0%)	46 (75.4%)	77 (63.1%)	
Tumor size								0.037
≤2 cm	175 (56.1%)	12 (46.2%)	17 (50.0%)	15 (50.0%)	12 (29.3%)	26 (42.6%)	58 (47.5%)	
>2 cm	137 (43.9%)	14 (53.8%)	17 (50.0%)	15 (50.0%)	29 (70.7%)	35 (57.4%)	64 (52.5%)	
Macroscopic type								0.151
0-I (Protruded)	30 (9.6%)	4 (15.4%)	2 (5.9%)	2 (6.7%)	3 (7.3%)	2 (3.3%)	4 (3.3%)	
0-IIa (Elevated)	35 (11.2%)	2 (7.7%)	3 (8.8%)	4 (13.3%)	4 (9.8%)	3 (4.9%)	6 (4.9%)	
0-IIb (Flat)	34(10.9%)	2(7.7%)	6(17.6%)	0(0.0%)	5(12.2%)	3(4.9%)	17(13.9%)	
0-IIc (depressed)	148 (47.4%)	14 (53.8%)	17 (50.0%)	17 (56.7%)	20 (48.8%)	33 (54.1%)	59 (48.4%)	
0-III (excavated)	65 (20.8%)	4 (15.4%)	6 (17.6%)	7 (23.3%)	9 (22.0%)	20 (32.8%)	36 (29.5%)	
Ulcer								0.385
Absence	168 (53.8%)	13 (50.0%)	15 (44.1%)	14 (46.7%)	17 (41.5%)	26 (42.6%)	54 (44.3%)	
Presence	144 (46.2%)	13 (50.0%)	19 (55.9%)	16 (53.3%)	24 (58.5%)	35 (57.4%)	68 (55.7%)	
Invasion depth								<0.001
M	175 (56.1%)	9 (34.6%)	18 (52.9%)	17 (56.7%)	13 (31.7%)	20 (32.8%)	50 (41%)	
SM1	55 (17.6%)	6 (23.1%)	3 (8.8%)	6 (20.0%)	10 (24.4%)	6 (9.8%)	23 (18.9%)	
SM2	82 (26.3%)	11 (42.3%)	13 (38.2%)	7 (23.3%)	18 (43.9%)	35 (57.4%)	49 (40.2%)	
LVI								0.001
Absence	287 (92.0%)	23 (88.5%)	30 (88.2%)	28 (93.3%)	29 (70.7%)	48 (78.7%)	101 (82.8%)	
Presence	25 (8.0%)	3 (11.5%)	4 (11.8%)	2 (6.7%)	12 (29.3%)	13 (21.3%)	21 (17.2%)	
Perineural invasion								0.012
Absence	305 (97.8%)	26 (100.0%)	32 (94.1%)	28 (93.3%)	38 (92.7%)	56 (91.8%)	110 (90.2%)	
Presence	7 (2.2%)	0 (0.0%)	2 (5.9%)	2 (6.7%)	3 (7.3%)	5 (8.2%)	12 (9.8%)	
LNM								<0.001
Negative	293 (93.9%)	24 (92.3%)	31 (91.2%)	26 (86.7%)	21 (51.2%)	49 (80.3%)	111 (91.0%)	
Positive	19 (6.1%)	2 (7.7%)	3 (8.8%)	4 (13.3%)	20 (48.8%)	12 (19.7%)	11 (9.0%)	
scope of LND								0.881
D1	28 (9.0%)	3 (11.5%)	3 (8.8%)	2 (6.7%)	4 (9.8%)	8 (13.1%)	9 (7.4%)	
D1+	59 (18.9%)	5 (19.2%)	9 (26.5%)	7 (23.3%)	13 (31.7%)	12 (19.7%)	26 (21.3%)	
D2	225 (72.1%)	18 (69.2%)	22 (64.7%)	21 (70.0%)	24 (58.5%)	41 (67.2%)	87 (71.3%)	
Number of LND	21.92 ± 9.99	19.27 ± 5.96	21.82 ± 8.96	23.87 ± 11.09	24.39 ± 10.73	21.70 ± 7.46	23.24 ± 9.54	0.279

LND, lymph node dissection.

### Pattern of LNM rate according to different PUC levels

**Figure 1:**
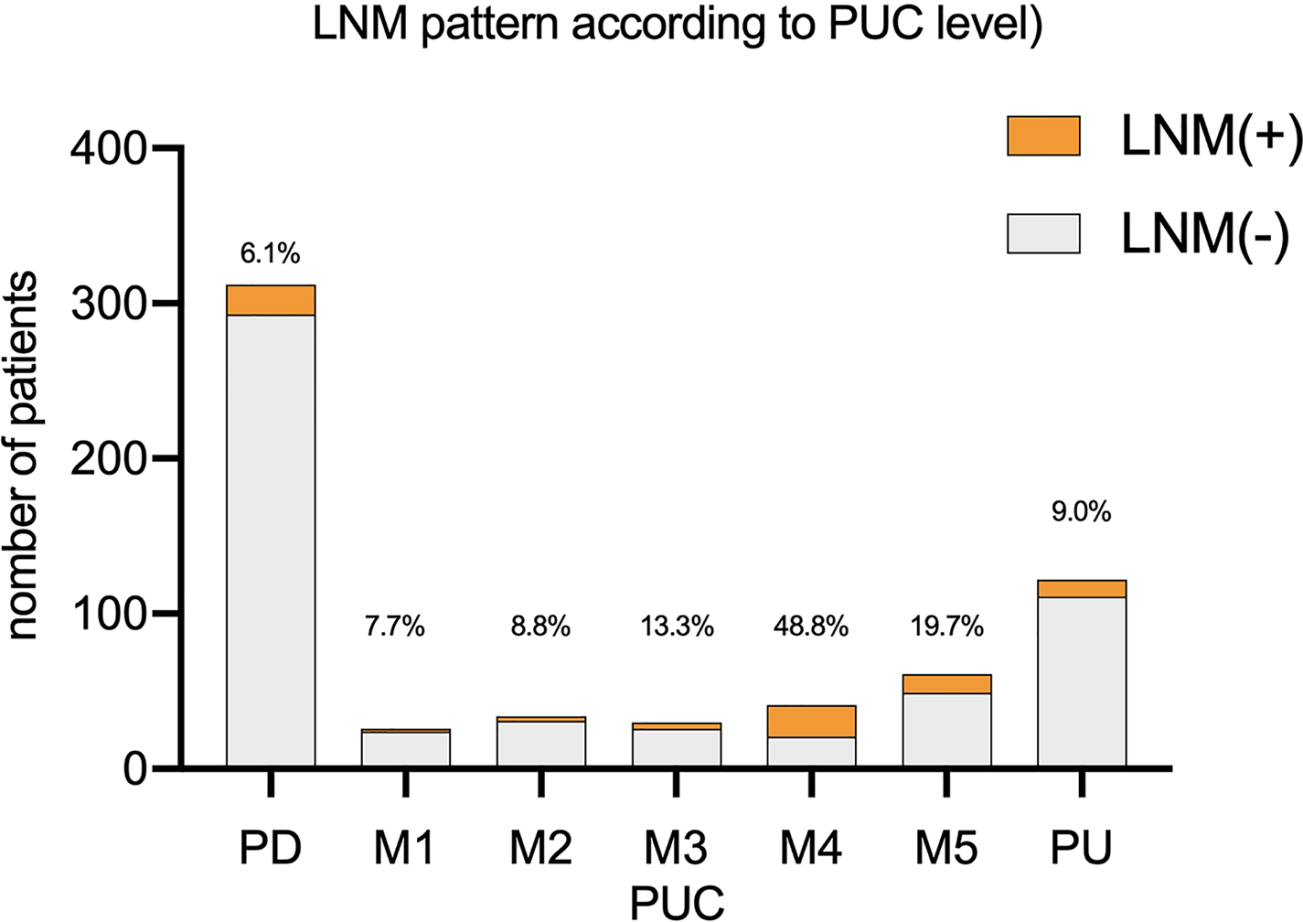
Distribution of LNM according to PUC level.

Comparison between each two groups showed that LNM rate was higher in group M4(M4 vs. PD: *p* < 0.001, M4 vs. M1: *p* = 0.010, M4 vs. M2: *p* = 0.004, M4 vs. M3: *p* = 0.038, M4 vs. M5: *p* = 0.040, M4 vs. PU: *p* < 0.001, all *p* value corrected by Bonferroni method). LNM rate of M5 was higher than PD (*p* = 0.009 after Bonferroni correction). While the LNM rate of M5(19.7%) was higher than PU (9.0%), there was no statistics difference between the two groups. Comparison between other each two groups also showed no significant difference (*p* > 0.05 after Bonferroni correction). Such results suggested that we should be more cautious about recommendations for mixed type lesions with PUC level over 60% after ESD. LNM rate according to PUC level was showed in Figure 1.

### Univariate and multivariate analysis of LNM risk factors in EGC

Based on the results above, to identify the clinicopathological predictive factors of LNM, univariate and multivariate logistic regression analyses were performed. The univariate analysis showed that larger tumor size (OR 4.015; 95% CI 2.245–7.179; *p* < 0.001), presence of ulcer (OR 2.038; 95% CI 1.212–3.424; *p* = 0.007), submucosa invasion to SM2 (OR 7.809; 95% CI 3.952–15.431; *p* < 0.001), presence of LVI (OR 18.082; 95% CI 10.201–32.052; *p* < 0.001), presence of perineural invasion (OR 2.973; 95% CI 1.261–6.843; *p* = 0.009), PUC level [(M4:OR 14.687; 95% CI 6.812–31.666), (M5:OR 3.777; 95% CI 1.725–8.267), *p* < 0.001] was significantly associated with LNM rate (Table 3). Based on enter method multivariate analysis, tumor size over 2 cm (OR 3.157; 95% CI 1.581–6.303; *p* = 0.001), submucosa invasion to SM2 (OR 2.869; 95% CI 1.262–6.523; *p* = 0.012), presence of LVI (OR 12.648; 95% CI 6.246–25.611; *p* < 0.001) and PUC level M4 (60% < PUC ≤ 80%) (OR 12.205; 95% CI 4.791–31.088; *p* < 0.001) significantly predicted LNM in EGC (Table 4).

**Table 3:** Risk of LNM in EGC according to clinicopathological characteristics.

Variable	Total *n* (%)	LNM negative *n* (%)	LNM positive *n* (%)	Univariate OR (95% CI)	*p*
Age (years)					0.573
≤60	202 (32.3%)	177 (31.9%)	25 (35.2%)	1	
>60	424 (67.7%)	378 (68.1%)	46 (64.8%)	0.862 (0.531,1.447)	
Sex					0.073
Male	429 (68.5%)	387 (69.7%)	42 (59.2%)	1	
Female	197 (31.5%)	168 (30.3%)	29 (40.8%)	1.591 (0.958,2.640)	
Tumor location					0.693
Upper third	48 (7.7%)	43 (7.7%)	5 (7.0%)	1	
Middle third	156 (24.9%)	141 (25.4%)	15 (21.1%)	0.915 (0.314,2.662)	
Lower third	422 (67.4%)	371 (66.8%)	51 (71.8%)	1.182 (0.448,3.122)	
Tumor size					<0.001
≤2 cm	315 (50.3%)	299 (53.9%)	16 (22.5%)	1	
>2 cm	311 (49.7%)	256 (46.1%)	55 (77.5%)	4.015 (2.245,7.179)	
Macroscopic type					0.758
0-I (Protruded)	47(7.5%)	42(7.6%)	5(7.0%)	1	
0-IIa (Elevated)	57(9.1%)	50(9.0%)	7(9.9%)	1.176(0.348,3.978)	
0-IIb (Flat)	67(10.7%)	60(10.8%)	7(9.9%)	0.980(0.291,3.298)	
0-IIc (depressed)	308(49.2%)	277(49.9%)	31(43.7%)	0.940(0.346,2.552)	
0-III (excavated)	147(23.5%)	126(22.7%)	21(29.6%)	1.400(0.497,3.945)	
Ulcer					0.007
Absence	307 (49.0%)	283 (51.0%)	24 (33.8%)	1	
Presence	319 (51.0%)	272 (49.0%)	47 (66.2%)	2.038 (1.212,3.424)	
Invasion depth					<0.001
M	302 (48.2%)	18 (52.9%)	11 (15.5%)	1	
SM1	109 (17.4%)	3 (8.8%)	11 (15.5%)	2.969 (1.248,7.063)	
SM2	215 (34.3%)	13 (38.2%)	49 (49.0%)	7.809 (3.952,15.431)	
LVI					<0.001
Absence	287 (92.0%)	23 (88.5%)	30 (88.2%)	1	
Presence	25 (8.0%)	3 (11.5%)	4 (11.8%)	18.082 (10.201,32.052)	
Perineural invasion					0.013
Absence	305 (97.8%)	26 (100.0%)	32 (94.1%)	1	
Presence	7 (2.2%)	0 (0.0%)	2 (5.9%)	2.937 (1.261,6.843)	
PUC level					<0.001
PD	312 (49.8%)	293 (52.8%)	19 (26.8%)	1	
M1	26 (4.2%)	24 (4.3%)	2 (2.8%)	1.285 (0.282,5.848)	
M2	34 (5.4%)	31 (5.6%)	3 (4.2%)	1.492 (0.418,5.328)	
M3	30 (4.8%)	26 (4.7%)	4 (5.6%)	2.372 (0.751,7.496)	
M4	41 (6.5%)	21 (3.8%)	20 (28.2%)	14.687 (6.812,31.666)	
M5	61 (9.7%)	49 (8.8%)	12 (16.9%)	3.777 (1.725,8.267)	
PU	122 (19.5%)	111 (20.0%)	11 (15.5%)	1.528 (0.705,3.314)	
Scope of LND					0.799
D1	57 (9.1%)	51 (9.2%)	6 (8.5%)	1	
D1+	131 (20.9%)	114 (20.5%)	17 (23.9%)	1.268 (0.472,3.403)	
D2	438 (70.0%)	390 (70.3%)	48 (67.6%)	1.528 (0.426,2.567)	
Number of LND					0.394
≤22	399 (63.7%)	357 (64.3%)	42 (59.2%)	1	
>22	227 (36.3%)	198 (35.7%)	29 (40.8%)	1.245 (0.752,2.061)	

LND, lymph node dissection.

**Table 4:** Multivariate analysis.

Variable	OR (95% CI)	*p*
Tumor size		0.001
>2 cm	3.157 (1.581,6.303)	
Invasion depth		0.012
SM2	2.869 (1.262,6.523)	
LVI		<0.001
Presence	12.648 (6.246,25.611)	
PUC level		<0.001
M4	12.205 (4.791,31.088)	

### Comparison of LNM rates in EGC according to indications for ESD

Mixed type lesions were regrouped into four groups: G1 (*n* = 34, 5.4%), G2 (*n* = 39, 6.2%), G3 (*n* = 52, 8.3%) and G4 (*n* = 67, 10.7%). One case in PD (1/172, 0.6%) and one case in G2 (1/23,4.3%) who met with absolute ESD indications for differentiated EGC developed with LNM, while no statistical difference was found in cases which met the absolute indications or in cases who was out of the absolute ESD indications for differentiated EGC. No LNM occurred in patients who met the absolute ESD indications for undifferentiated EGC in group G3 (0 of 3), G4 (0 of 6) and PU (0 of 20). For cases beyond the absolute ESD indications for undifferentiated EGC, LNM rate was significant higher in G4 (G4 vs. PU: 44.9% vs. 10.8%, *p* < 0.001; G4 vs. G3: 44.9% vs. 18.0%, *p* = 0.002) (Shown in Tables 5, 6).

**Table 5:** Comparison of LNM between pure differentiated and differentiated predominant lesions according to ESD indications for differentiated EGC.

Group	PD	G1 (0% < PUC ≤ 25%)	G2 (25% < PUC < 50%)	*p*
	LNM (%)	LNM (%)	LNM (%)	
Ab	1 of 172 (0.6%)	0 of 11 (0%)	1 of 23 (4.3%)	0.304
Non-ab	18 of 140 (12.9%)	3 of 23 (13.0%)	4 of 16 (25.0%)	0.396

Lesions that fit for absolute indications for ESD of differentiated EGC. Non-ab: Lesions that do not fit for absolute indications for ESD of differentiated EGC.

**Table 6:** Comparison of LNM between pure undifferentiated and undifferentiated predominant lesions according to ESD indications for undifferentiated EGC.

Group	PU	G3 (50% ≤ PUC ≤ 75%)	G4 (75% < PUC < 100%)	*p*
	LNM (%)	LNM (%)	LNM (%)	
Ab	0 of 20 (0%)	0 of 3 (0%)	0 of 6 (0%)	-
Non-ab	11 of 102 (10.8%)	11 of 61 (18.0%)	22 of 49 (44.9%)	<0.001

Lesions that fit for absolute indications for ESD of undifferentiated EGC. Non-ab: Lesions that do not fit for absolute indications for ESD of undifferentiated EGC.

### Nomogram development and internal validation

**Figure 2:**
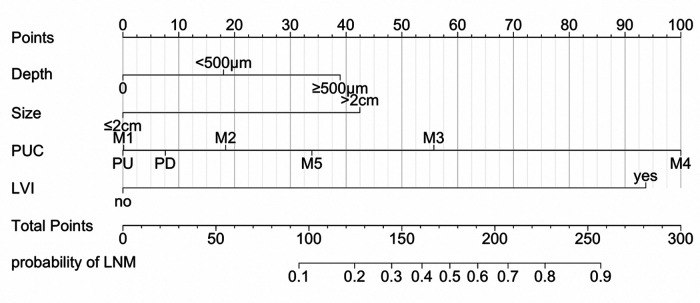
The nomogram for predicting LNM in EGC.

**Figure 3:**
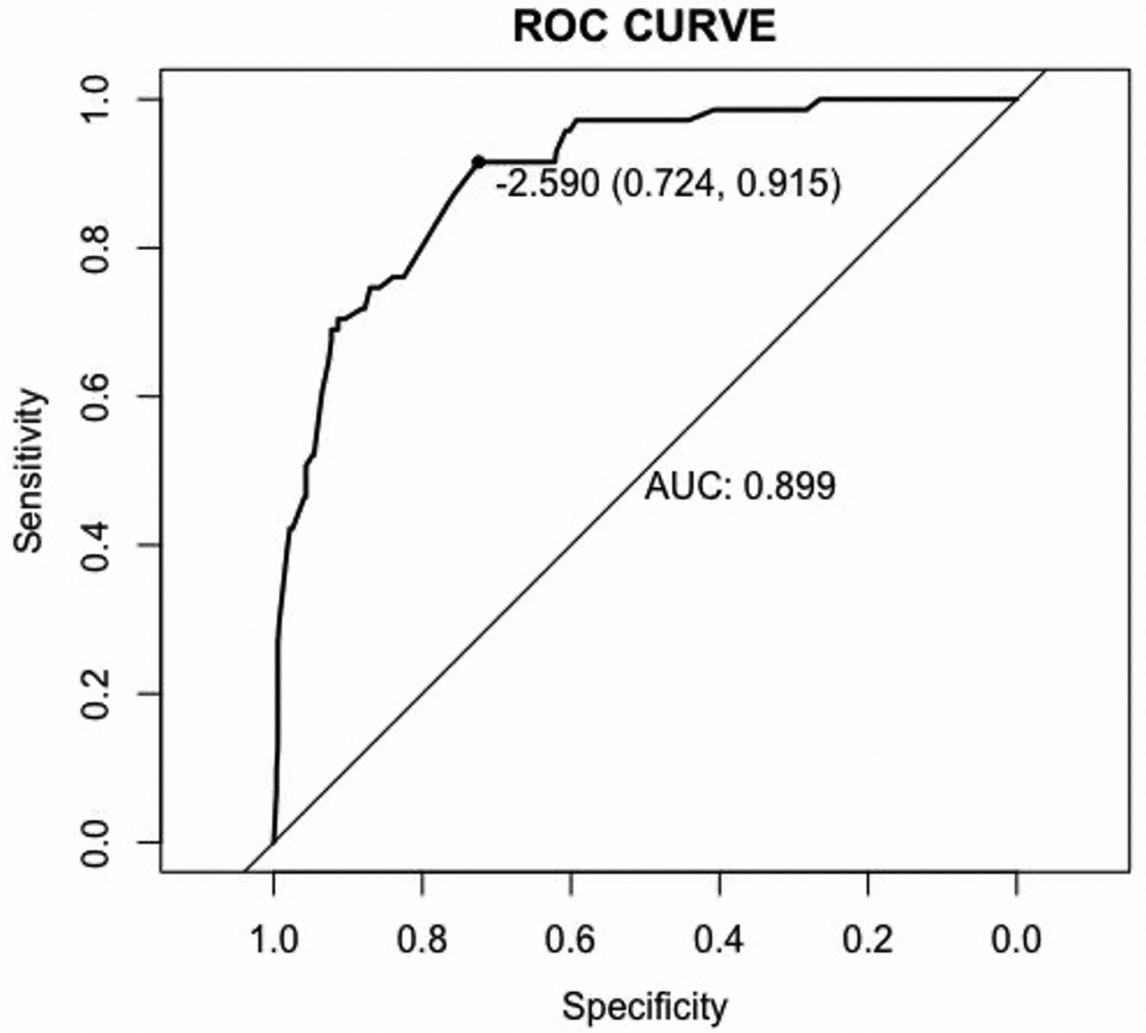
Receiver operating characteristic (ROC) curves of the model.

**Figure 4:**
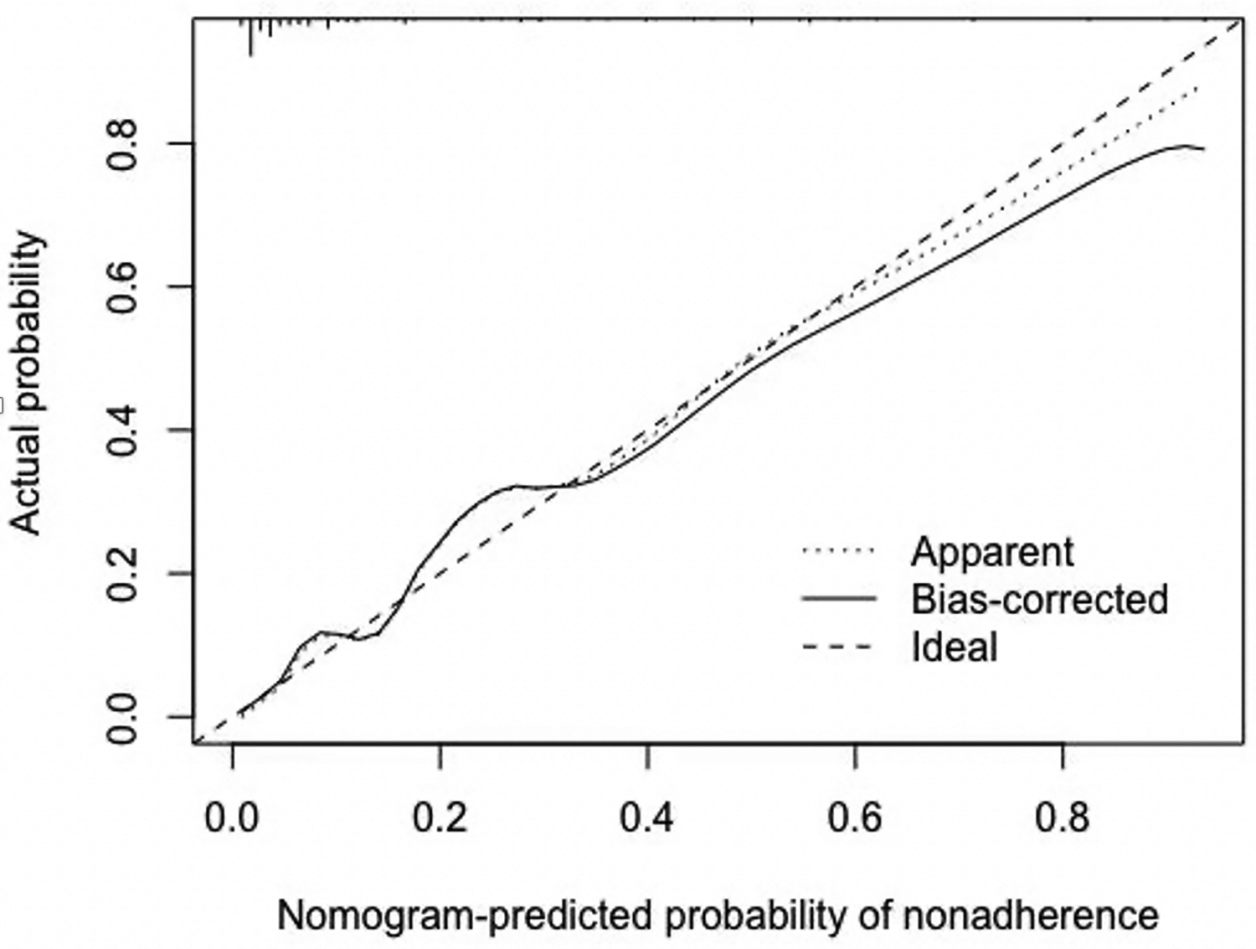
The internal calibration curve of the model.

Based on the results of multivariate logistic analysis, a nomogram was developed to predict LNM in EGC. Showed in Figure 2, the nomogram score was 39 for submucosa infiltration depth ≥ 500, 42.5 for tumor size > 2 cm, 100 for M4 PUC level and 93 for LVI, respectively. AUC of the model was 0.899 (range 0.724–0.915) (Figure 3). Predictive performance internal validation by Bootstrap method showed a consistency index of 0.899, the internal calibration curve showed optimal agreement between actual observations and model predictions (Figure 4). And a good fitting effect was demonstrated by Hosmer - Lemeshow test (*χ*² = 7.187, *p* > 0.05).

## Discussion

ESD is widely accepted as a treatment method for patients with EGC who meet with the appropriate indications. Presence of LNM is a crucial factor concerned by clinicians when arranging the more appropriate therapy for EGC patients. Several parameters such as LVI, tumor size, invasion depth, presence of ulcer had been confirmed to be related to LNM in EGC. Several previous studies showed that EGC with mixed histological type was more aggressive (24–26), but none of the studies focused on the PUC levels, which might be different on clinicopathological features and related to LNM in EGC. Since proportion of each histologic type varies widely in different EGC lesions, we investigated clinicopathological features of EGC according to different PUC levels.

In our study, LNM rate of mixed type EGC was higher than rate of pure histology type EGC (16.6% vs. 6.1%, *p* < 0.01), which supports some of the previous reports (24–26). However, we revealed a pattern that clinicopathological features including LNM rate differ according to PUC level. LNM rate between PD and mixed type groups (M1, M2 and M3) showed no statistical difference, while LNM rate for mixed type lesions with PUC level over 60% (M4, M5) was significantly higher than PD. In our study, with a limited sample size, LNM rate in M4 was statistically higher than and PU. While LNM rate of M5(19.7%) was higher than PU (9.0%), there was no statistics difference between the two groups after Bonferroni correction. Such results above suggested that we should be more cautious about recommendations for mixed type lesions with PUC level over 60% after ESD.

Since tumor size, invasion depth and LVI were all showed to be associated with LNM, we hypothesis that differences of these parameters between groups might have influence on forming of this LNM rate pattern. Therefore, including PUC level as a parameter, we further investigated risk factors of LNM in EGC. In line with previous studies, presence of ulcer, invasion depth, tumor size, presence of LVI and histological type were associated with LNM. For histological type, LNM was showed to be more prevalence in t mixed type EGC (19, 27–30). In our study, we further performed analysis between subgroups and made a step forward to locate that PUC level M4 was an independent risk factor of LNM in EGC. This might indicate a phenomenon that LNM risk does not always parallel with proportion of undifferentiated components.

We also explored feasibility of updated ESD indications for EGC patients according to different PUC levels. Between groups, no statistical difference of LNM rate was found in cases who met the absolute ESD indications for differentiated type EGC or undifferentiated type EGC patients, respectively. For cases that beyond the range of absolute indications for undifferentiated EGC, LNM rate was significant higher in G4(75% < PUC < 100%). Based on our results, we think that PUC level could be one of the factors considered when we further explore the ESD indications for EGC.

It is a tendency that ESD is being accepted by more EGC patients and more studies would be carried out to expand indications of ESD for EGC in the future. At the same time, the necessity for remedial surgery after endoscopic resection basically depends on the presence of lymph node metastasis. Therefore, it is important to predict LNM in EGC. Based on results of logistic regression, we developed a nomogram, aimed to provide some reference for EGC patients who received endoscopic resection. The parameters in the nomogram (tumor size, invasion depth, PUC level and LVI status) could be evaluated in ESD specimens. The nomogram could provide some reference for the risk of LNM in patients after ESD, especially in patients who received non-curative resection. It might be helpful for clinicians to recommend a better postoperative treatment plan to EGC patients.

Limitations exist in this study. Firstly, though cut off values determination and pathological evaluation methods were based or developed from previous studies, there is still no better consensus achieved by us. Secondly, since this study was based on data from a single center, sample size was limited and external verification could not be performed to test the nomogram. Thus, further studies are still needed to verify the performance of our nomogram and to explore ESD indications for mixed type EGC.

## Data Availability

The raw data supporting the conclusions of this article will be made available by the authors, without undue reservation.
